# Machine-Based Hand Massage Ameliorates Preoperative Anxiety in Patients Awaiting Ambulatory Surgery

**DOI:** 10.1097/jnr.0000000000000432

**Published:** 2021-04-12

**Authors:** Cheng-Hua NI, Li WEI, Chia-Che WU, Chueh-Ho LIN, Pao-Yu CHOU, Yeu-Hui CHUANG, Ching-Chiu KAO

**Affiliations:** 1 MS, RN, Supervisor, Department of Nursing, Center for Nursing and Healthcare Research in Clinical Practice Application, Wan Fang Hospital, Taipei Medical University, and Adjunct Assistant Professor, School of Nursing, College of Nursing, Taipei Medical University, Taiwan, ROC; 2 MD, PhD, Assistant Professor, Graduate Institute of Injury Prevention and Control, College of Public Health, Taipei Medical University, and Attending Physician, Division of Neurosurgery, Department of Surgery, Wan Fang Hospital, Taipei Medical University, Taiwan, ROC; 3 MD, PhD, Assistant Professor, School of Medicine, College of Medicine, Taipei Medical University, and Attending Physician, Department of Otolaryngology, Wan Fang Hospital, Taipei Medical University, Taiwan, ROC; 4 PhD, PT, Associate Professor, Master Program in Long-Term Care, College of Nursing, Taipei Medical University, Taiwan, ROC; 5 MS, RN, Head Nurse, Department of Nursing, and Center for Nursing and Healthcare Research in Clinical Practice Application, Wan Fang Hospital, Taipei Medical University, and Adjunct Instructor, School of Nursing, College of Nursing, Taipei Medical University, Taiwan, ROC; 6 PhD, RN, Professor, School of Nursing, College of Nursing, Taipei Medical University, and Center for Nursing and Healthcare Research in Clinical Practice Application, Wan Fang Hospital, Taipei Medical University, Taiwan, ROC; 7 MS, RN, Executive Director of Community Medicine, Center for Nursing and Healthcare Research in Clinical Practice Application, Wan Fang Hospital, Taipei Medical University, and Adjunct Assistant Professor, School of Nursing, College of Nursing, Taipei Medical University, Taiwan, ROC; 8 Contributed equally as corresponding author.

**Keywords:** ambulatory surgery, anxiety, hand massage, preoperative, State-Trait Anxiety Inventory (STAI)

## Abstract

**Background:**

Hand massage therapies have been used to relieve anxiety and pain in various clinical situations. The effects of machine-based hand massage on preoperative anxiety in ambulatory surgery settings have not been evaluated.

**Purpose:**

This prospective study was designed to investigate the effect of machine-based hand massage on preoperative anxiety and vital signs in ambulatory surgery patients.

**Methods:**

One hundred ninety-nine patients aged 18 years and older who were scheduled to receive ambulatory surgery were recruited from the Taipei Municipal Wanfang Hospital in Taipei City, Taiwan. The patients were assigned randomly to the experimental group (*n* = 101), which received presurgical machine-based hand massage therapy, and the control group (*n* = 98), which received no intervention. The patients in both groups completed the Spielberger State-Trait Anxiety Inventory short form at preintervention (baseline) and postintervention.

**Results:**

Within-group comparisons of Spielberger State-Trait Anxiety Inventory short form scores showed significant decreases between preintervention and postintervention scores in the experimental group (44.3 ± 11.2 to 37.9 ± 8.7) and no significant change in the control group. Within-group comparisons of vital signs revealed a significant increase in mean respiration rate between baseline and postintervention in both groups (both *p*s < .05). Blood pressure was found to have decreased significantly only in the control group at postintervention (*p* < .05). No significant preintervention to postintervention change in pulse was observed in either group.

**Conclusions:**

The findings of this study indicate that machine-based hand massage reduces anxiety significantly in patients awaiting ambulatory surgery while not significantly affecting their vital signs.

## Introduction

Anxiety is recognized as a normal response of patients during the preoperative period. Anxiety has been identified as the most common negative emotion in patients awaiting diagnostic procedures such as coronary angiography and may contribute to complications during these procedures (H. Li et al., 2016; Mei et al., 2017). Investigators have suggested that the preoperative waiting period in the hospital is an extended period during which patients tend to worry about their impending surgery/procedure (Gilmartin & Wright, 2008; Mitchell, 2003). Anxiety has been shown to affect the physiological and psychological status of patients adversely. In addition, preoperative anxiety has been identified as a predictor of postoperative pain (Brand et al., 2013; Kunikata et al., 2012) and has been associated with delayed recovery (Mavros et al., 2011). Furthermore, reducing anxiety in preoperative patients is important because undue anxiety may also interfere with patients' ability to learn and remember important postoperative home care instructions (Gilmartin & Wright, 2008).

The application of hand massage therapy in outpatients waiting for ambulatory surgery or diagnostic procedures has been evaluated in several previous studies (Brand et al., 2013; Kunikata et al., 2012; Nazari et al., 2012). Lower anxiety levels of anxiety have been reported in patients receiving hand massage therapy than in their peers who received routine nursing care only (Brand et al., 2013). Kunikata et al. (2012) measured autonomic activity and psychological indicators before and after a hand massage intervention, reporting a significant decrease in heart rate after the intervention, indicating a decrease in autonomic and sympathetic nerve activity and a promotion of relaxation. Furthermore, hand massage has been reported to reduce preoperative anxiety significantly in patients scheduled to receive ophthalmology surgery under local anesthesia (Kim et al., 2001; Nazari et al., 2012).

Various other applications of hand massage therapies have been used to relieve anxiety and pain in different clinical situations. For example, adding intraoperative hand reflexology during a minimally invasive conscious surgery for varicose veins was found to reduce patients' anxiety and the duration of postoperative pain (Hudson et al., 2015). Similarly, hand reflexology was shown to decrease anxiety in a clinical trial designed to study the effects of this intervention in patients waiting to receive a coronary angiography (Mobini-Bidgoli et al., 2017). Both hand massage and hand-holding methods delivered by nurses have been shown to reduce anxiety and to promote a sense of affinity in the patient toward the massage giver, with the one-on-one contact involved in massage associated with a positive anxiolytic effect (Kunikata et al., 2012).

Massage machines have been applied in clinical settings for years (Q. Li et al., 2019; Yoshida et al., 2014). Although the effects of manual hand massage on preoperative anxiety in ambulatory surgery patients have been studied previously (Kim et al., 2001; Nazari et al., 2012), to the best of our knowledge, the effect of machine-based hand massage on preoperative anxiety in ambulatory surgical settings has not yet been evaluated. Machine-based hand massage may be as effective as physical massage in ambulatory surgery patients awaiting ambulatory surgical procedures under local anesthesia. Therefore, the aim of this study was to evaluate the effects of machine-based hand massage on preoperative anxiety and vital signs in ambulatory surgery patients.

## Methods

### Study Design and Sample

This prospective study was conducted in the waiting area outside the surgical suites at Taipei Municipal Wanfang Hospital, Taipei, Taiwan. Patients awaiting ambulatory surgery aged 18 years and older were recruited using a random sampling approach from the Departments of Otolaryngology, Dermatology, Urology, and General Surgery from July 2016 to June 2017. Patients with catastrophic illness cards, a mental illness, or a malignant tumor and those who were scheduled for hand surgery were excluded. Screened patients waiting for surgery were asked to fill out a situational anxiety questionnaire and were instructed on how to complete it. Those who agreed to complete the questionnaire and were willing to participate in the study were enrolled as participants. All of the included patients (*n* = 199) were randomly assigned to either the experimental group (*n* = 101), which received presurgical hand massage therapy, or the control group (*n* = 98), which received no intervention.

### Ethical Considerations

This study was approved by the Taipei Medical University Joint Institutional Review Board (approval number: N201512022). All of the participants provided signed informed consent to participate.

### Intervention Procedure

The patient waiting area for ambulatory surgery was air-conditioned and had individually partitioned spaces and sofa-type seating with backs, allowing the patients to wait for surgery in comfort. The vital signs of patients in both groups were measured, and their basic demographic information was collected by the research assistants. The experimental equipment consisted of sphygmomanometers, ear thermometer, and hand massage machines. Participants in both groups received general, routine surgical nursing care. All of the participants were scheduled to undergo local surgery with local anesthesia and were discharged from the hospital on the same day.

To ensure the consistency of hand massage, the participants in the experimental group each received 15 minutes of hand massage administered using two identical commercial hand massagers (Breo iPalm 520 acupressure hand massager with heat compression; Chino, CA, USA) on each hand simultaneously. The hand massager temperature was set at 39°C. The machine was covered with plastic wrap for each use, which was replaced after each use to prevent cross-contamination. Patients in both groups completed the postintervention situational anxiety questionnaire after all of the experimental group participants had received hand massages.

### Anxiety Evaluation

The short form of the Spielberger State-Trait Anxiety Inventory (STAI-S; Marteau & Bekker, 1992) was used to measure anxiety. The STAI-S is a self-administered scale suitable for use with adults and teenagers. This instrument comprises the situational anxiety and trait anxiety subscales, comprising 20 questions scored using a 4-point Likert scale. The STAI-S has been used in clinical settings to diagnose anxiety and to differentiate it from depression. A Chinese version of the STAI-S has been used widely in Taiwan to evaluate responses to various therapies (Lee et al., 2017; Wu et al., 2017). For the 10-item situational anxiety subscale, 1 represents *not at all*, 2 represents *somewhat*, 3 represents *moderately so*, and 4 represents *very much so*. For the 10-item trait anxiety subscale, reverse scoring is used, with scores ranging from 1 representing *very much so* to 4 representing *not at all*. Higher STAI-S scores indicate greater anxiety. The test–retest reliability is .89 for the Chinese version of the STAI-S.

### Statistical Analysis

Continuous variables are presented as mean ± standard deviation (*SD*) by group and are compared using the two-sample *t* test or Mann–Whitney *U* test for nonnormally distributed variables. Categorical variables are presented as counts and percentages and compared using the chi-square test or Fisher's exact test. A two-sample *t* test was used to detect between-group differences at baseline and postintervention and to identify changes between baseline and postintervention. Paired *t* test was employed to compare within-group differences between baseline and postevaluation. A nonparametric Mann–Whitney *U* test was applied to compare the differences between the two groups for nonnormally distributed data. All of the assessments were two-sided, and *p* < .05 was used to determine statistical significance. All statistical analyses were performed using SAS Version 9.4, Windows NT version (SAS Institute, Inc., Cary, NC, USA).

## Results

The 101 experimental group participants received hand massage therapy before surgery, whereas the 98 participants in the control group received standard care only. The baseline characteristics of the participants are shown in Table 1. The distribution of baseline characteristics was similar between the two groups, with the exception of working status. Mean age was 41.1 ± 14.0 and 41.3 ± 15.7 years for the control group and experimental group, respectively. No significant differences were found between the two groups in terms of gender, education, religion, marital status, living status, smoking status, alcohol consumption, being accompanied during surgery, knowing the length of surgery, or surgery history. However, working status was significantly different between the two groups (Table 1).

**Table 1.** Baseline Demographics and Clinical Characteristics of the Participants

Variable	Control (*n* = 98)		Hand Massage Therapy (*n* = 101)		*p*^a^
	Mean	*SD*	Mean	*SD*	
Age (years)	41.1	14.0	41.3	15.7	.93
Height (cm)	166.7	8.9	165.9	9.4	.32
Weight (kg)	67.3	14.6	65.3	14.5	.27
Pain score ^b^	0.1	0.3	0.1	0.2	.12
Pulse rate (bpm)	79.1	13.6	77.9	13.2	.47
Respiration rate (bpm)	16.4	1.9	16.5	1.7	.88
Systolic pressure (mmHg)	132.9	22.7	133.2	21.9	.73
Diastolic pressure (mmHg)	78.9	13.2	77.4	13.4	.38

	*n*	%	*n*	%	
Gender					.35 ^c^
Male	54	55.1	49	48.5	
Female	44	44.9	52	51.5	
Education					.44 ^c^
Elementary	4	4.1	10	9.9	
Junior	7	7.1	7	6.9	
Senior	24	24.5	32	31.7	
Faculty	14	14.3	11	10.9	
Bachelor	35	35.7	30	29.7	
Master or above	14	14.3	11	10.9	
Religion					.63 ^c^
Buddhist	35	35.7	36	35.6	
Catholic/Christian	48	49.0	54	53.5	
Muslim	15	15.3	11	10.9	
Marital status					.20 ^d^
Unmarried	35	35.7	42	41.6	
Married	54	55.1	56	55.5	
Divorced	6	6.1	3	2.9	
Widowed	3	3.1	0	0	
Living status					.51 ^d^
Alone	14	14.3	11	10.9	
With family	80	81.6	88	87.1	
With friends	4	4.1	2	2.0	
Working status					.04 ^d,^*
No	21	21.4	33	32.7	
Yes	68	69.4	57	56.4	
Retired	6	6.1	11	10.9	
Cannot work with disease	3	3.1	0	0	
Smoking status					.29 ^c^
No	65	66.3	77	76.2	
Yes	25	25.5	19	18.8	
Quit smoking	8	8.2	5	5.0	
Alcohol consumption ^e^					.11 ^d^
No	70	71.4	82	82.0	
Yes	21	21.4	16	16.0	
Quit drinking	7	7.2	2	2.0	
Accompanied during surgery					.06 ^c^
No	47	48.0	35	34.7	
Yes	51	52.0	66	65.3	
Know the expected duration of surgery ^e^					.49 ^c^
No	65	67.7	63	63.0	
Yes	31	32.3	37	37.0	
Surgery experience ^e^					.28 ^c^
No	31	31.6	39	39.0	
Yes	67	68.4	61	61.0	

^a^ Mann–Whitney *U* test. ^b^ The scale of pain score: 1 to 10. ^c^ Chi-square test. ^d^ Fisher's exact test. ^e^ Numbers may not equal to total sample size due to missing value.

*p < .05.

Mean changes in STAI-S scores between baseline and postintervention are shown in Table 2. Mean STAI-S scores at baseline were 44.3 ± 11.2 in the experimental group and 42.6 ± 9.7 in the control group. Statistically significant differences were found in mean STAI-S scores at postintervention, which were 37.9 ± 8.7 in the experimental group and 43.8 ± 9.9 in the control group (*p* < .05). Changes in STAI-S scores from baseline (preintervention) to postintervention were also significantly different between the two groups (1.1 ± 5.7 vs. −6.4 ± 7.5; *p* < .05). In addition, an analysis of the within-group comparison revealed that hand massage significantly decreased STAI-S scores (*p* < .05; Table 2).

**Table 2.** Mean Changes in STAI-S Scores, by Group

STAI-S Score	Control (*n* = 98)		Hand Massage Therapy (*n* = 101)		*p*
	Mean	*SD*	Mean	*SD*	
Baseline	42.6	9.7	44.3	11.2	.26 ^a^
Postintervention	43.8	9.9	37.9	8.7	< .001^a,^*
Change from baseline to postintervention	1.1	5.7	−6.4	7.5 ^b,†^	< .001^c,^*

*Note.* STAI-S = Spielberger State-Trait Anxiety Inventory short form.

^a^*T* test. ^b^ Paired *t* test. ^c^ Mann–Whitney *U* test.

*Significant difference between groups, *p* < .05.

^†^Significant difference between preintervention and postintervention, *p* < .05.

Mean changes in vital signs between baseline and postintervention are shown in Table 3. No significant differences were found between the groups in terms of mean changes in preintervention and postintervention vital signs, including pulse, respiration rate, systolic pressure, and diastolic pressure. In addition, within-group comparison analysis revealed that postintervention respiration rates had significantly increased from baseline in both groups (*p* < .05), whereas blood pressure was found to have decreased significantly after the intervention only in the control group (*p* < .05; Table 3).

**Table 3.** Mean Changes in Vital Signs, by Group

Vital Sign	Control (*n* = 98)		Hand Massage Therapy (*n* = 101)		*p*^a^
	Mean	*SD*	Mean	*SD*	
Pulse (bpm)					
Baseline	79.1	13.6	77.9	13.2	.47
Postintervention	78.9	13.7	78.9	11.5	.91
Change from baseline to postintervention	−0.2	9.2	1.0	7.8	.31
Respiration rate (bpm)					
Baseline	16.4	1.9	16.5	1.7	.88
Postintervention	17.5	4.5	17.3	1.7	.36
Change from baseline to postintervention	1.0	4.3 ^b,^*	0.8	1.7 ^b,^*	.59
Systolic pressure (mmHg)					
Baseline	132.9	22.7	133.2	21.9	.73
Postintervention	129.9	18.4	131.9	18.0	.28
Change from baseline to postintervention	−2.9	11.6 ^b,^*	−1.4	11.9	.58
Diastolic pressure (mmHg)					
Baseline	78.9	13.2	77.4	13.4	.38
Postintervention	76.9	10.6	77.8	11.6	.69
Change from baseline to postintervention	−2.1	7.5 ^b,^*	0.4	9.1	.08

^a^ Mann–Whitney *U* test. ^b^ Paired *t* test.

*Significant difference between preintervention and postintervention, *p* < .05.

## Discussion

The results of this study show a significant difference in preintervention/postintervention STAI-S scores between the two groups, indicating that the reduction in anxiety was significantly larger in the experimental group than in the control group. In addition, the within-group comparison revealed that the intervention significantly reduced STAI-S scores in the experimental group, whereas no significant preintervention/postintervention difference was found in STAI-S scores in the control group. In terms of vital sign measurements, the within-group comparison revealed that the postintervention mean respiration rate was significantly higher in both groups and that postintervention blood pressure significantly decreased in the control group only. No significant postintervention change in pulse was found in either group.

Anxiety in the experimental group was significantly lower after the intervention. In addition, although the control group participants received routine preoperative care only, their vital signs were not significantly different from their peers in the experimental group. These results echo those of other studies on the effects of person-delivered hand massage therapy on preoperative and preprocedure anxiety. Although all of the patients in Brand et al. (2013) reported decreased anxiety before entering the operating suite, only those who had received the hand massage intervention exhibited significantly lower anxiety as measured using visual analog scores. The nurses who cared for those patients found that hand massage and reduced anxiety made it easier to set up intravenous lines in the preoperative area because of facilitated vasodilation and patients' overall level of comfort. In another study that used the STAI to measure anxiety in preoperative patients before and after a hand massage intervention (Kunikata et al., 2012), STAI scores decreased significantly after the hand massage. However, whereas patients' blood pressure and heart rates decreased after the intervention, their respiration rate did not. In contrast, in a clinical trial on pain and anxiety after cesarean section (Saatsaz et al., 2016), pain and anxiety as well as the physiological measures of blood pressure and respiration rate were reduced significantly after a hand massage intervention. As evidence remains unclear, the relationship between receiving a hand massage and changes in postoperative vital signs should be further investigated. Potential reasons for a direct relationship are that the improved blood flow and quality of sleep resulting from massage improves perceived anxiety and that massage therapy increases parasympathetic nervous system activity, releasing neurotransmitters and/or reducing cortisol levels.

Hand massage used as an alternative or complementary therapy may be implemented as a simple and effective nursing intervention in preoperative settings (Braithwaite & Ringdahl, 2017; Brand et al., 2013). In addition to the effects on patients' preoperative/preprocedure anxiety, providing hand massage therapy is of potential benefit to nurses as well. Nurses who have administered hand massage procedures to preoperative patients have reported gaining increased awareness of patients' psychological, emotional, and medical needs (Brand et al., 2013). Hand massage may be delivered by nurses or trained volunteers, with the results of studies on volunteer-administered intervention suggesting that family members and other caregivers may be easily trained to administer hand massage therapies both before a procedure and after hospital discharge (Gensic et al., 2017). Using a machine rather than a person to provide hand massages not only reduces the workload of caregivers but also provides consistent massages. Further side-by-side pretest and posttest studies related to the clinical application of machine-based hand massage in ambulatory settings are necessary to compare the effectiveness of machine-based hand massages with hand massages administered by nurses.

### Strengths and Limitations

The strengths of this study include the random selection of ambulatory surgery patients and the administration of hand massage therapy consistently across patients, which enabled the effects of the intervention on anxiety and vital signs to be evaluated. One limitation was the lack of a side-by-side comparison of the results of machine-based hand massage with the results of nurse-administered hand massage, which would indicate the comparative efficacy of the two approaches. A second limitation was that the hospital personnel (mostly nurses) who provided routine care for the two groups of ambulatory surgery patients were not blinded to the group assignments, introducing the risk of performance bias. A third limitation was that, because nurse-administered hand massage is subject to variation among different nurses and different patients, an additional study is needed to compare the two methods and to evaluate the effectiveness of machine-based hand massage therapy in a larger, multicenter population.

### Conclusions

Machine-based hand massage was shown to significantly reduce anxiety in ambulatory surgery patients. However, no dramatic effects of machine-based hand massage on vital signs were observed. Further studies are necessary to confirm the results of this study and to evaluate the effects of machine-based hand massage in comparison with the effects of nurse-delivered hand massage.
